# Bone Formation in Grafts with Bio-Oss and Autogenous Bone at Different Proportions in Rabbit Calvaria

**DOI:** 10.1155/2020/2494128

**Published:** 2020-02-19

**Authors:** Yeon Jung Kim, Carlos Eduardo Takeshi Saiki, Karoline Silva, Carlos Kiyoshi Moreira Massuda, Ana Paula de Souza Faloni, Paulo Henrique Braz-Silva, Debora Pallos, Wilson Roberto Sendyk

**Affiliations:** ^1^Department of Implantology, University of Santo Amaro-UNISA, São Paulo, SP, Brazil; ^2^Department of Health Sciences, Implantology Post-Graduate Course, University Center of Araraquara-UNIARA Dental School, Araraquara, SP, Brazil; ^3^Department of Stomatology, School of Dentistry, University of São Paulo—USP, São Paulo, SP, Brazil; ^4^Laboratory of Virology, Institute of Tropical Medicine of São Paulo, School of Medicine, University of São Paulo—USP, São Paulo, SP, Brazil

## Abstract

**Background:**

The aim of this study was to assess the volumetric stability and bone formation in grafts with Bio-Oss and autogenous bone at different proportions in rabbit calvaria. *Material and Methods*. Ten rabbits received four titanium cylinders in their calvaria and randomly divided into the following groups: Group I: Bio-Oss (100%), Group II: Bio-Oss (75%) + autogenous bone (25%), Group III: Bio-Oss (50%) + autogenous bone (50%), and Group IV: autogenous bone (100%). After twelve weeks, the animals were euthanized, and samples were collected for clinical and histological analysis.

**Results:**

Clinical analysis showed that Groups I (90.43 ± 8.99) and II (90.87 ± 7.43) had greater dimensional stability compared to Group IV (*P*=0.0005). Histologically, Groups I, II, and III showed areas of bone formation with particles of biomaterial remaining in close contact with the newly formed bone. However, there were no significant differences between the groups regarding the newly formed bone area.

**Conclusion:**

It was concluded that the use of Bio-Oss either alone or associated with the autogenous bone at a proportion of 25% showed superior dimensional stability compared to the use of autogenous bone in the proposed experimental model.

## 1. Introduction

Several biomaterials and procedures have been used for bone augmentation in the attempt to increase the long-term success of dental implants [1–3]. An adequate bone volume is one of the critical factors for a successful osseointegration and long-term retention of endosseous dental implants [4–6].

Autogenous bone is considered the gold standard for reconstructive techniques due to its biocompatibility and having osteogenic, osteoconductive, and osteoinductive properties. However, it presents some disadvantages such as the need for additional surgical donor site, associated morbidity, and rapid resorption rate [2–4, 7, 8].

Thus, several biomaterials have been developed in an attempt to reduce the use of or even replace the autogenous bone graft in reconstructive surgeries. Among these, Bio-Oss—a deproteinized bovine bone—has been used in dentistry for bone augmentation procedures due to its osteoconductive properties [9]. Previous studies have demonstrated effectiveness, safety, and high success rates regarding the quality and quantity of bone formation with Bio-Oss in grafting procedures [1, 10, 11]. In addition, adequate results have been already demonstrated in terms of integration without local hosting and low reabsorption [12–14].

The use of autogenous bone in combination with the deproteinized bovine bone is believed to be a better method because of its osteogenic property [10–16]. Despite the extensive literature, preclinical studies comparing volumetric stability and bone formation in bone augmentation procedures using Bio-Oss and autogenous bone grafts are scarce. Moreover, there is no consensus in the literature on the ideal proportion of Bio-Oss and autogenous bone graft. The aim of this study was to compare the volumetric stability and bone formation in grafts with Bio-Oss and autogenous bone at different proportions in rabbit calvaria. The hypothesis of this study was that the proportion of the amount of autogenous bone and Bio-Oss in the graft could influence the volumetric stability and the grater new bone formation.

## 2. Materials and Methods

This present study has been approved by the Animal Experiment Committee of University of Santo Amaro (CEUA 11/2011).

A total of ten New Zealand rabbits weighing between 3.0 and 4.0 kg, aged between 11 and 15 months, were kept in individual cages under controlled temperature (22–25°C) and received a standard laboratory diet and water *ad libitum*.

### 2.1. Surgical Procedure

For all surgical procedures, the animals were initially anesthetized with ketamine 10% (40 mg/kg body wt.) (Ketamin S., Crisfarma, Sergipe, Brazil) and xylazine 2% (8 mg/kg body wt.) (Virbaxyl 2%, Virbac, São Paulo, Brazil), both intramuscularly, before local infiltration of 2% lidocaine with norepinephrine (Alphacaine, DFL, Rio de Janeiro, Brazil). In addition, the animals were maintained on intravenous hydration with saline solution 0.9% during surgery.

**Figure 1:**
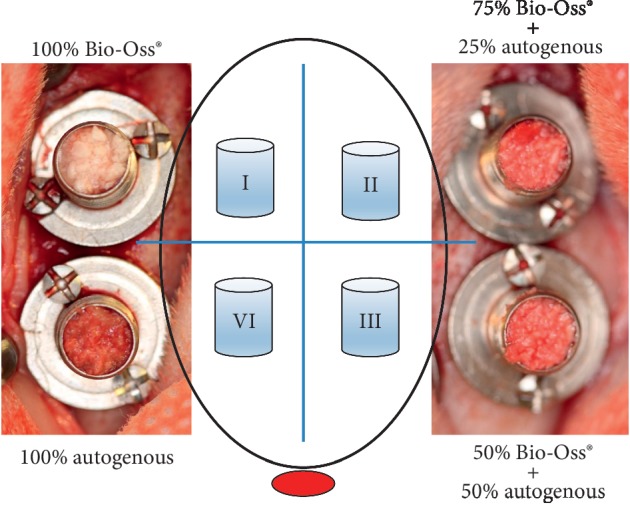
Schematic drawing of the cylinders distributed into four groups according to the grafts placed in the rabbit caldarium.

Incision was made in the skin of the calvaria region, and the tissues were dissected to expose the calvarial bone. Each animal received four titanium cylinders of 5 mm in diameter × 5 mm in height (Conexão®, São Paulo, Brazil) attached with two screws of 1.5 mm in height × 1.5 mm in diameter (Neodent®, Curitiba, Brazil). Decortication of the internal region of the cylinder was made with a 1.1 mm diameter drill under constant irrigation with saline solution 0.9%. The cylinders were randomly divided into four groups according to the grafts (Figure 1) as follows:

  Group I: anorganic bovine bone graft (100% Bio-Oss)
  Group II: anorganic bovine bone graft (75% Bio-Oss) + 25% autogenous bone
  Group III: anorganic bovine bone graft (50% Bio-Oss) + 50% autogenous bone
  Group IV: 100% autogenous bone

The autogenous bone graft was obtained from the iliac crest. The lateral surface of the posterior iliac crest was exposed by a skin incision, and the tissues were dissected to show the iliac crest bone. Next, a cortical-cancellous bone graft was harvested and then separated. Autogenous bone graft and anorganic bovine bone graft (particle size of 1-2 mm; Bio-Oss, Geistlich Pharmaceutical, Wolfhausen, Switzerland) were proportionally mixed according to the experimental groups.

The cylinders were filled with the biomaterial according to the experimental groups (Figure 1) and covered before the wounds were sutured with 4-0 nylon threads (Ethicon, Johnson & Johnson, São José dos Campos, Brazil).

Postoperatively, the animals were medicated with antibiotics enrofloxacin 2.5% (0.4 ml/kg body wt.), enrofloxacin 10% (Vencofarma, Londrina, Brazil), and Flotril® (Schering Plough, São Paulo, Brazil) by subcutaneous injections for seven days as well as with analgesic meloxicam intramuscularly for seven days (0.01 ml/kg body wt.) (Maxicam®, OuroFino, São Paulo. Brazil).

### 2.2. Calculation of Bone Graft Volume

**Figure 2:**
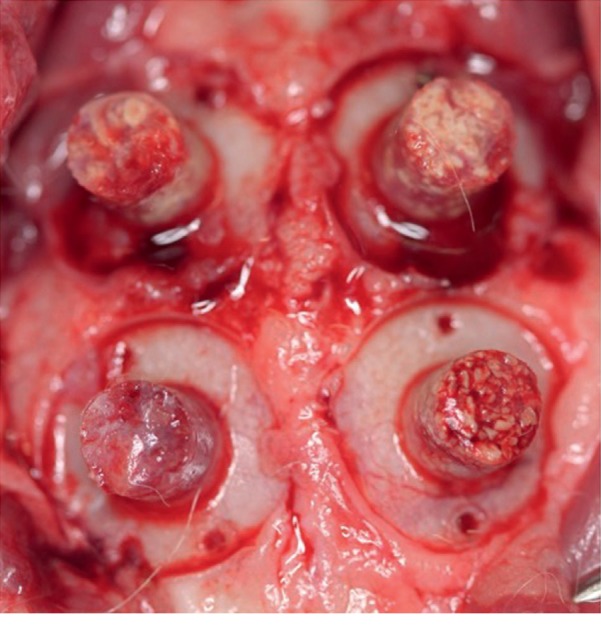
Clinical image of the bone grafts in the rabbit calvarium 12 weeks after the surgery.

After 12 weeks, all animals were euthanized with an overdose of anaesthetics. The calvaria were surgically reopened, and the screw caps of the cylinder were removed (Figure 2). Next, the height of the bone graft was measured by one blinded calibrated examiner, who used a PDT Sensor Probe™ (Type U.S, Zila Pharmaceuticals, Phoenix, USA) in four sites of each sample.

The volume of the bone graft was calculated according to the following formula:

(1) $$\begin{array}{c} V=\;\pi \;\times \;r^{2}\;\times \;h, \end{array}$$

where *V* is the volume, *π* is a mathematical constant = 3.1415, *r* is the cylinder radius = 2.5 mm, and *h* is the mean height of the formed tissue graft.

### 2.3. Tissue Processing and Histomorphometric Analysis

The calvarial bone of each animal was carefully removed and then sectioned together with the four cylinders. The samples were fixed in 10% formalin for 48 hours prior to routine histological processing and paraffin embedding. Histological serial sections with 5 *μ*m thickness were cut in the sagittal plane (Jung Supercut 2065 Leica, Chicago, IL, USA), and hematoxylin and eosin (HE) was used for staining them. Histological slides were analysed by using a light microscope (LEICA Microsystems GmbH, Wetzlar, Germany), and images were captured with a digital camera (Leica DFC 300 FX, Wetzlar, Germany) at a 40x magnification. The digital images were exported for histomorphometric analysis, which was performed by a calibrated blinded examiner using specific software (ImageJ, https://imagej.nih.gov/ij/) in a standardized manner.

### 2.4. Statistical Analysis

Data analysis was performed by using the GraphPad Prism software (GraphPad Software Inc., La Jolla, CA, USA). In relation to volume data, Shapiro–Wilk's test was used to assess data normality, and Friedman's test was used for comparison between the groups.

After testing the normal distribution of the histomorphometric data with Kolmogorov–Smirnov test, statistical significance was assessed by using one-way ANOVA followed by Tukey's post hoc test for multiple comparisons between the groups at a statistical significance of *P* < 0.05.

## 3. Results

### 3.1. Volume Maintenance of the Bone Graft

All groups had samples integrated to the calvarium of the animals, without signs of infections.

In relation to volume, Groups I and II showed resorption less than 10% of the total volume, whereas Group IV showed resorption greater than 30%. Thus, there was a greater volume maintenance in Groups I and II compared to Group IV (*P*=0.0005) (Table 1).

### 3.2. Histomorphometric Analysis

**Figure 3:**
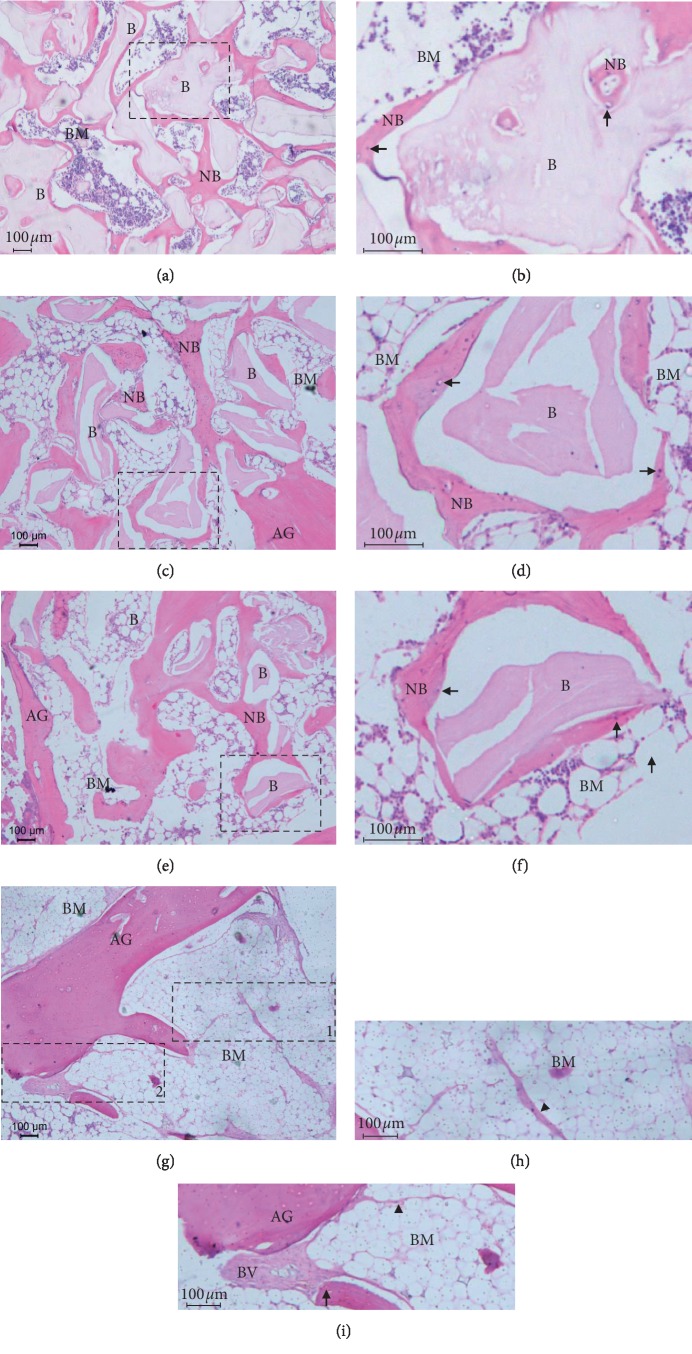
Photomicrographs showing bone formation in different groups: (a, b) Group I (100% Bio-Oss), (c, d) Group II (75% Bio-Oss + 25% autogenous bone), (e, f) Group III (50% Bio-Oss + 50% autogenous bone), and (g, h, i) Group IV (100% autogenous bone). Hematoxylin and eosin staining of all groups. Bars: 100 *μ*m. Residual particles of biomaterial (B), newly formed bone (NB), bone marrow (BM), autogenous bone graft (AG), osteocyte (arrow), and blood vessels (BV). Extracellular matrix of bone marrow seems to be replaced by adipose tissue (arrowhead). Dotted squares in (a), (c), and (e) represent the areas observed at higher magnification in (b), (d), and (f), respectively. Dotted rectangles (1) and (2) in (g) represent the areas observed at higher magnification in (h) and (i), respectively.

There was no inflammatory process in all histological sections evaluated. The grafted material was integrated into the pre-existing bone, demonstrating a good bone-healing pattern with normal mineralization of the neo-formed bone. In addition, Groups I, II, and III showed regions of bone neo-formation interspersed with residual biomaterial in which the remaining biomaterial particles were in contact with bone apposition on their surface. The bone marrow space varied in volume along the bone, presenting either greater or lesser amounts (Figure 3). However, there were no significant differences regarding the bone area between the groups (Table 2).

## 4. Discussion

This study was carried out in rabbits based on a methodology already applied by our research team in which titanium cylinders were attached to the calvaria of rabbits with two screws to create and stabilize an area to be filled with the biomaterial [17, 18]. This simulates a clinical situation in which there is no wall with vascularization pathway, which is similar to that found in maxillary sinus lift surgery or in which there is a critical bone defect with only one remaining bone wall, permitting the comparison of four different ratios of Bio-Oss and autogenous bone in the same animal.

Among the biomaterials, Bio-Oss has been widely used in bone augmentation procedures due to its high success rate and predictability. Earlier studies have tested different ratios of the autogenous bone associated with Bio-Oss in bone augmentation procedures, ranging from the addition of an undefined proportion of the autogenous bone [10, 19, 20] to ratios of 2 : 1 [21], 1.5 : 1 [22], 1 : 1 [12, 15, 16, 23], 1 : 2 [15], and 1 : 4 [15, 24–26]. The purpose of this approach is to add viable osteoblasts to improve bone induction and bone formation. However, there is still no consensus regarding the ideal proportion of autogenous bone and Bio-Oss to optimize bone formation [13].

In our study, the data demonstrated that the autogenous bone alone undergoes faster resorption and remodeling compared to any other mixture, showing that the volume reduction in Group IV was statistically significant compared to Groups I, II, and III (Table 1). Some studies using only autogenous graft for bone augmenting procedures showed unpredictable reduction in volume [4, 27, 28].

The volume stability of the graft is an important factor for implant survival, and the proportion of Bio-Oss has a significant influence on the graft. These findings are consistent with other studies using only Bio-Oss as a graft material as they reported higher dimensional maintenance compared to the autogenous bone in the short and long terms [26, 29, 30]. Also, Bio-Oss demonstrated high volume maintenance and new bone formation [12, 13, 30].

In the present study, the histomorphometric analysis revealed similar rate of the newly formed bone between the groups (Table 2). These results indicate that quantity and quality of the newly formed bone were the same in the groups not influenced by the percentage of autogenous bone. Also, the newly formed bone in close contact with the residual particles in the groups using Bio-Oss suggests that this biomaterial induces bone formation without any influence on the bone graft resorption due to its stability and osteoconductive property.

The volume stability was significantly influenced by the proportion of Bio-Oss in the bone graft. The addition of Bio-Oss in any proportion would be beneficial for the maintenance of the grafted region, resulting in long-term implant survival.

## 5. Conclusions

One can conclude that the use of Bio-Oss alone or associated with the autogenous bone in the proportion of 25% showed superior dimensional stability compared to the use of autogenous bone alone in the proposed experimental model.

## Figures and Tables

**Table 1:** Volume maintenance of the bone graft.

	Group I	Group II	Group III	Group IV
Bone graft volume (%)	92.68 ± 6.05	90.87 ± 7.43	82.28 ± 8.57	67.02 ± 17.89^*∗*^

Mean ± SD (%); ^*∗*^*p*=0.0005; Group I = Group II > Group IV.

**Table 2:** Histomorphometric analysis of the areas of the newly formed bone.

	Group I	Group II	Group III	Group IV	*p*
Newly formed bone (mm^2^)	2.44 ± 0.80	2.24 ± 0.85	2.63 ± 0.72	3.33 ± 0.66	0.25
Residual particles (mm^2^)	2.70 ± 0.79	2.37 ± 0.60	2.03 ± 0.41	0.00^*∗*^	<0.0001

Mean ± SD.

## Data Availability

The data used to support the findings of this study are available from the corresponding author upon request.
